# Examining non-LTR retrotransposons in the context of the evolving primate brain

**DOI:** 10.1186/s12915-017-0409-z

**Published:** 2017-08-11

**Authors:** Sara B. Linker, Maria C. Marchetto, Iñigo Narvaiza, Ahmet M. Denli, Fred H. Gage

**Affiliations:** Salk Institute for Biological Studies, La Jolla, CA 92037-1002 USA

## Abstract

Researchers have long sought to understand the genetic basis of the cognitive differences between primates, with particular focus on the human brain. Although all mutational types have worked in concert with evolutionary forces to generate the current human brain, in this review we will explore the impact of mobile elements, specifically non-LTR retrotransposons. Non-LTR retrotransposons have contributed coding and regulatory sequences to the genome throughout evolution. During primate evolution there have been multiple waves of LINE retrotransposition as well as the birth of new mobile elements such as the SINEs Alu and SVA and we will explore what kinds of impacts these may have had on the evolving human brain.

## Genetic complexity of the human condition

The human species has developed art, literature, science, technology, agriculture, and grant cycles, all aspects of the human condition that are not observed in any other species of primate. Unique qualities of the human brain such as a relatively large cortical volume, surface area, and altered connectivity are often cited as key structural reasons for this increased complexity with respect to physiology. A yet unresolved question is the identification of the genetic modifications that underlie these physiological and cognitive complexities. Although all forms of mutation work together with selection and drift to produce the ever-dynamic phenotype, we will explore here the contribution of the repetitive portion of the genome to the evolution of the human brain, specifically that of non-LTR retrotransposons (RTs). By exploring the impact of RTs in disease, non-neuronal systems, and neuronal systems of model organisms we can gain insight into the still unresolved question of what role RTs might play in modifying neuronal function both throughout primate evolution and within the lifetime of a single individual human.

## An evolutionary history of RTs during the expansion of the primate brain

RTs are present in most eukaryotic genomes and make up almost 40% of the human genome [1]. There are two major classes of active RTs in humans: long interspersed elements (LINEs) and short interspersed elements (SINEs). Of the RTs that are active in primates, there are two SINEs (SINE/Variable number tandem repeat/Alu (SVA) and Alu) and one LINE ( LINE-1 (L1)) that are commonly active in humans. The most ancient clades of eukaryotic RTs (GENIE, CRE, and R4) can be traced back at least 600 million years ago (mya) with the most ancient element identified within the primitive eukaryote *Giardi lamblia* [2–4]. Although the origin of non-LTR RTs can be traced to near the split of prokaryotes from eukaryotes, RTs did not expand to near present-day levels until the beginning of mammalian evolution, with the majority of currently fixed elements having inserted in the early primate genome between 12 and 40 mya [5–8]. While primate-specific RTs were beginning to populate the genome, these early ancestors were adapting to the changing conditions that would eventually generate the modern human, and with it the modern human brain.

**Fig. 1.**
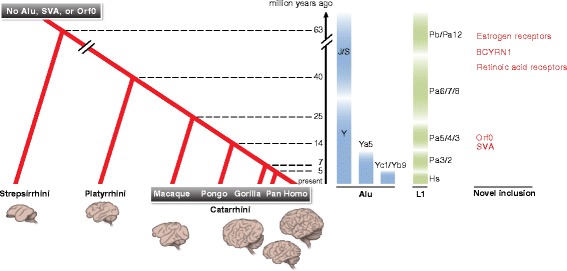
Phylogenetic timeline of primate evolution. The major branches represent Strepsirrhini, Platyrrhini, and five genera of Catarrhini (Macaca, Pongo, Gorilla, Pan, and Homo), with branch points denoting the hypothesized most recent common ancestor (million years ago). Drawings underneath each branch represent the increased brain volume and cortical folding for each genus. The waves of retrotransposition that have been predicted to occur within the past 63 million years in primates are shown for two major families of retrotransposons; Alu (*blue*) and L1 (*green*). For each wave of retrotransposition the names of common active subfamilies are noted. New additions to the genome driven by each wave of retrotransposition are noted in *red* and coinciding changes in brain structure are noted in *black*

To provide context for the following sections related to the functional impact of these RTs, we provide below a temporal map that intersects the integration timing of primate-specific RT subfamilies with major physiological changes that represent landmarks in the evolution of the primate brain (Fig. 1). It should be noted that this intersection is not meant to indicate that RTs directly caused each of these events, but the hope is to provide a general context with which to explore the connection between RTs and neuronal function.

For example, during the Eocene (~35–65 mya), early prosimians proliferated. These ancestors of modern-day lemurs and tarsiers had a brain that was relatively small compared to their body mass and with relatively little gyrification [9, 10]. The early prosimian genome supported retrotransposition of ancient families of L1 (L1M, L1PA, and L1PB) and HERV. This period of time also witnessed the explosion of the extant Alu subfamilies AluJ and AluS, which had previously existed only as the smaller fragments FLAM and FRAM and which are present in all known primates. The split between platyrrhines and catarrhines occurred around 35 mya [11]. Platyrrhines are similar to prosimians in that they have a relatively small brain size as well as shallower sulci, or folds in the brain, than catarrhines; both of these features can impact higher cognitive functions [9, 12, 13]. The central sulcus, a prominent cortical fold, began to increase in size compared to the overall cortical surface area after the split of lesser apes from catarrhines [14, 15]. Around 25 mya AluY and the L1PA8, L1PA7, and L1PA6 families began to take over [5, 8]. At that time, the split occurred between hominoids and cercopithecoids [11]. In RT history, this was the time when SVAs first originated and the reign of the L1PA4/5 subfamilies began [16]. This time was also associated with an increase in size of the frontal cortex. The final point on our RT-brain map is the time of the largest increase in primate brain size, which occurred within the past ~5 my [17]. After the Pan-Homo split the human brain departed from the norm of allometric scaling [9]. The RTs that flourished during this period in *Homo* were the subfamilies L1Hs, AluYa5, AluYb8, and SVA, and these elements are still the most active elements in the human genome. The current estimated rates of germline insertion are ~1 in 20 births for Alu, 1 in 270 births for L1, and 1 in 916 births for SVA [18–20].

As with any mutation these insertions were more often than not likely to have neutral or even deleterious impacts on the population. However, in the odd case that a new insertion provided a selective advantage, the question becomes whether the proliferating subfamily of RT contained unique properties that enabled a more efficient and directed impact on neuronal evolution.

## The susceptibility and resilience of brain genes to retrotransposition

**Fig. 2.**
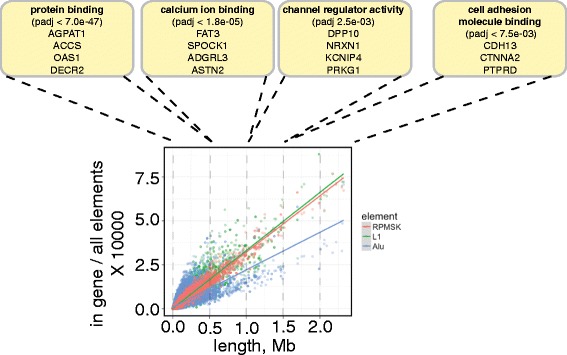
RT copy number as a function of gene length. RTs consisted of all repeat masked elements, only L1, or only Alu. Gene length was calculated as transcription start to transcription stop. The element count was normalized by the total number of elements across all genes. Genes were then subdivided into four bins as noted by diagonal lines and the top Gene Ontology term (molecular function) was noted along with the Benjamini corrected *p* value and top genes [93]. Note that, similar to findings from previous studies, the largest genes are commonly cell adhesion molecules, channel genes, and calcium ion binding genes that are important for neuronal function [23, 24]

The impact of a RT is largely dependent on its genomic location. Other than a small subset of regions (for example, the NF1 gene), there has been no strong evidence to suggest that there are specific hotspots of RT integration [21]. However, at random chance, RTs are more likely to reside within the introns of long genes. Importantly, long genes are enriched for genes that are expressed in the brain, specifically expressed within neuronal populations, and involved in synapse formation, cell adhesion, and other neuronal-specific processes [22–24]. Given their length, these neuronal genes have an increased susceptibility to have retrotransposons insert within the transcriptional boundaries (Fig. 2). However, the presence of a RT does not equal the function of that element; therefore, it is important to examine whether retrotransposition within these long introns has a significant impact on neuronal function. Although this information is not directly known for the human brain, the cross-species phenomenon of purifying selection of RTs within genes indicates that selection may be acting on these elements, although the influence of genetic drift can not be ruled out [25–27]. Furthermore, studies examining the comparative density of RTs within long versus short introns have identified that germline RTs are reduced in prevalence within genes and as they become closer to an exon, and this effect is more stringent for RTs in the antisense orientation [27, 28]; however, it is unclear whether this effect is consistent in long introns. As these reports are limited to examining the impact of germline RTs, the question remains open as to what extent insertions into introns directly impact neuronal function.

One known consequence of intronic RTs that is heightened within the brain is RNA editing of the primate-specific Alu RT. Brain-related genes such as those that are important for neuronal excitability have an increased propensity to undergo RNA editing due to their enrichment of intronic Alu, which is a primary recruiting signal for the ADAR enzyme that catalyzes these A to I transitions [29–34]. These RT-driven editing events have also been shown to alter the function of a neuron and this is reviewed in detail in Rosenthal and Seeburg [29]. For example, in human cells, an AluJ element is present in an intron of the GABA receptor GABRA3. This element promotes A-to-I editing of a neighboring exon, converting an isoleucine to methionine, which then results in altered sensitivity and deactivation of GABRA3 receptors [32, 35, 36]. The functional impact of this AluJ element establishes the proof-of-principle that Alu RTs can supply an additional level of functional modulation to neuronal genes through A-to-I editing. Given the millions of primate-specific Alu loci and their propensity to undergo editing, it is likely that at least a small subset of these elements impact neuronal function beyond just the GABRA3 receptor.

RTs also bring regulatory elements to gene regions; a concept that was initially posed by Britten and Davidson [37] and which was recently reviewed comprehensively in Chuong, Elde, and Feschotte [38]. For example, RTs that were active before the split of Platyrrhines from Catarrhines distributed DNA binding elements that are important for development throughout the primate genome [39]. The functionality of these primate-specific elements remains unclear as even the impact of RTs that integrated within early mammalian lineages is still under debate. For example, work from the Noonan lab showed that mammalian neocortical enhancers were not likely the result of transposons having immediately taken on a functional role upon insertion as the repeats with enhancer activity did not show signatures of conservation [40]. This finding potentially opposes the Britten and Davidson hypothesis; however, the MER130 family seems to be at least one exception [41]. The Bejerano lab identified that MER130 elements present within a highly specific set of p300-bound neocortical enhancers indeed displayed enhancer activity using an in vitro assay [41]. Importantly, these two reports are not mutually exclusive as they indicate that although RTs may display neocortical enhancer activity, as a general rule they are not directly advantageous sans additional mutations.

Further reports on the impact of RTs in development focus on the retinoic acid response which is an integral component of neurogenesis [42–46]. In humans, more than 90% of retinoic acid response elements (DR2) are derived from Alu elements that were active both before the split from prosimians (AluS) and after the split between platyrrhines and catarrhines (AluY) [47, 48]. Although it is unclear whether these DR2-containing Alus are important in human neurogenesis, they do display function in the human stem cell. With the addition of retinoic acid, DR2-containing Alus are actively transcribed and subsequently broken down into small RNAs that are required for the proper regulation of human stem-cell proliferation [49, 50]. Given the importance of the retinoic acid response in neuronal differentiation [44–46], future studies could benefit from determining whether these primate-specific DR2-containing Alus are analogously important for human neurogenesis. An additional binding signal, the estrogen response element for estrogen receptor-alpha, is similarly present largely due to the expansion of the ancient AluS subfamily [51, 52]. Examination of these elements in vitro showed that they are indeed bound by ER-alpha and functionally modulate local transcription [51, 53]. However, a study examining estrogen response in breast cancer showed that Alus were not preferentially bound in this cell line, indicating that caution must be used when attempting to link the presence of a binding site with the functional utility of that site [54].

A recent discovery from our lab suggests that transposons may also have made previously unknown contributions to the proteome [55]. Primate L1s encode a third open reading frame, ORF0, in the antisense orientation. ORF0 dates back to the L1PA8 subfamily, which was active after the split between Catarrhines and Platyrrhines. What makes this ORF particularly interesting is the presence of splice donor sites within its coding sequence. These donor sites act in concert with splice acceptor sites within proximal exons to generate fusion proteins. Thus, L1s can generate insertion-site specific proteins in Catarrhines, including humans. Around 3000 ORF0 loci exist in the human and chimp genomes and their transcription is enriched in pluripotent cells. Considering that a number of ORF0 fusions are associated with neuronal genes, it is possible that ORF0 may contribute to primate-specific properties of the brain. Which of the fusion events have evolved functions is currently under investigation.

## Advantages of RTs within the nervous system

The contribution of RTs to the health and normal function of the human brain is still readily debated. Progress is inherently slowed by the same impediments that plague all genomics research, such as small effect sizes and complex phenotypes. However, disease studies that have been reviewed extensively elsewhere [56, 57] have established links between RTs and neurological phenotypes. Although generalized contributions such as splicing, methylation, and A-to-I editing are known, experiments are needed that directly link specific RT loci to neuronal function and behavior. So far, examples of the advantageous nature of RTs in the human brain are limited, but two cases point to an evolved role as functional non-coding RNAs (ncRNA). For example, a composite Alu/L1 sequence within the SLC7A2 gene generates an ncRNA that is vital for human brain development and results in infantile encephalopathy when mutated [58]. Similarly, a monomeric Alu present after the split of prosimians and anthropoids now encodes the functional BCYRN1 ncRNA (aka Bc200) [59, 60]. The function of BCYRN1 is to aid protein synthesis in neuronal dendrites and, interestingly, this function has been replicated in the mouse genome through convergent evolution of mouse Bc1 from the rodent SINE B2 [60–63]. Future research should shed further light on these instances of advantageous adaptation of RTs. As increasing numbers of neurotypical individuals are sequenced, the RT component underlying normal human phenotypic variation should begin to be revealed and provide a library of locations to examine experimentally. Through these tools, we will begin to answer the lingering question: to what extent do RTs impact normal human phenotypic variation?

Although the focus of this review is on non-LTR RTs in humans, evidence from non-LTR RTs and DNA transposons in non-human species can provide helpful insight into the potential for mobile elements to impact neuronal function. One piece of evidence that non-LTR RTs can take on functional roles comes from a SINE that integrated over 170 mya and which currently functions as a tissue-specific enhancer in hypothalamic neurons [64]. Mobile elements can also impact on neuronal function by the generation of new genes such as POGZ, a Pogo element that when mutated can lead to microcephaly, intellectual disability, and autism spectrum disorders [65–68]. Furthermore, the immediate early gene, Arc, which is vital for long-term memory formation, is thought to have been generated from a Ty3/Gypsy LTR [69, 70]. These, as well as numerous other examples, indicate that mobile elements, including non-LTR RTs, have impacted the evolution of neuronal function in a way that is relevant to the human brain [71–75].

## Somatic retrotransposition in the human brain

Genomic changes in response to retrotransposition also persist on the much shorter scale of a single human lifetime. Over the past decade, interest in brain somatic retrotransposition has steadily risen, largely in response to the findings, including evidence from our lab, that RTs can mobilize in neural progenitor cells [76–78]. Interest was further buoyed by the repeated findings of genetic mosaicism in adult human neurons [79–82], including an increased number of RTs in the prefrontal cortex of individuals diagnosed with schizophrenia [83]. This has led to a consortium of 15 institutions, including our lab, gathering in the ‘Brain Somatic Mosaicism Network’ to tackle the issue of identifying the diverse types of mosaicism present within the human brain, including retrotransposition [84].

This continued interest from labs around the world has resulted in independent confirmation that retrotransposition is indeed a phenomenon acting not only on the timescale of human evolution but also throughout the development of a single human brain. Recent studies, including our own, using single-cell sequencing approaches have found that both L1 insertions and L1-mediated deletions are prevalent in the human brain [82, 85–87]. These studies estimate a rate of retrotransposition between ~0.6 and 13 somatic insertions per neuron [86, 87]. It is likely that the true rate of neuronal somatic retrotransposition in humans is somewhere between those two bounds, with 0.6 representing a lower bound due to methodological limits of detection and 13 being the upper bound limited by artifacts created during whole genome amplification. Importantly, since the experiments that calculated the lower bound have high rates of validation, it is reasonable to take 0.6 insertions per neuron as approximating a true minimum. Although 0.6 elements per neuron seems small, with approximately 86 billion neurons in the adult human brain, that comes to approximately 51 billion somatic retrotransposition events within a given individual. Even under a conservative mutational hypothesis, where a majority of new insertions are neutral, it is easy to imagine a scenario where these somatic events modulate a portion of functional heterogeneity within the human brain.

Importantly, mosaic mutations would have the potential to have a large effect on the cell that they reside in but relatively small effect on cells that are independent of that founder cell. Therefore, the more cells within a tissue rely on each other for proper function, the more likely it will be that a single mutation alters the function of that tissue. Considering the highly networked state of the brain, neurons are a particularly useful system to study the impact of somatic mosaicism since a small number of functional mutations could have far-reaching effects on neuronal circuitry. In fact, studies examining the impact of individual neurons in rodents have shown that disrupting the firing of a single neuron can affect rodent behavior [88, 89], indicating that the function of individual neurons can have a profound impact on behavioral diversity. If the clues from selection on RTs are any sign of their impact on function, then it is possible that new RT insertions can alter genetic and phenotypic heterogeneity within a brain within a single generation.

## Insights and outlook

Cognitive differences exist between humans and nonhuman primates that allow for the development of sophisticated behaviors such as language, self-awareness, symbolic thought, and cultural learning [88]. Although great advances have been made since the first discovery of RTs in humans, the functional contribution of RTs to these differences is still largely unknown. While previous efforts attempted to home in on RT function by examining signatures of selection, current efforts to increase our understanding are beginning to take advantage of high-throughput sequencing approaches to incorporate information from thousands of individuals as well as across multiple species. These future efforts will be aided by a more detailed and comprehensive approach to cataloguing and sharing this information. While RTs can influence the host independently of mobility, the number of active elements and their genomic locations will be instrumental in understanding their role in human biology, especially in somatic cells. In the future, we will have to move away from simply annotating transposable elements and take their context and activity state into consideration. Only a multi-species, comprehensive analysis and catalog of transposable elements will allow a true understanding of the influence of transposable elements on human brain evolution.

The prediction that RTs might have consequences for human brain development can inform experiments to be performed with new cell culture techniques, providing a powerful tool to probe the impact of RTs on human brain evolution. Currently, the vast amount of information available for comparative studies between humans and our closest relatives comes from DNA/RNA samples extracted from preserved (post-mortem) tissues. These samples do not always fairly represent the function of a region due to confounding effects of environment and development. Ideally, the identification of differences in genetic makeup between related species should be translated into phenotypic divergence in a controlled setting. Cell culture models utilizing neurons derived from non-human primate induced pluripotent stem cells could provide new insights into human adaptation features and could be genetically modified to determine the effects of individual loci of species-specific RTs. For example, in cell culture, RT loci that are predicted to have a functional impact can be mutated with gene editing technology (for example, CRISPR/Cas9 or the TALEN system), thereby enabling a direct study of the impact of RTs across neurons from different primate species. Work from our lab deriving induced pluripotent stem cells from primate lineages has begun to aid in these types of experiments [90–92]. Despite the findings presented here that clearly show that RTs have impacted the mammalian, primate, and human nervous system, the direct impact of RTs on the human brain currently remains under debate. Therefore, future studies, such as those using these induced pluripotent stem cell models, will help to define the role of RTs in the function of the human neuron.
